# Acoustic emission characteristics and damage evolution of different rocks under uniaxial compression conditions

**DOI:** 10.1038/s41598-024-54950-9

**Published:** 2024-02-20

**Authors:** Jianchun Ou, Enyuan Wang, Xinyu Wang, Bican Wang, Guoqing Zhu

**Affiliations:** 1 State Key Laboratory for Fine Exploration and Intelligent Development of Coal Resources, China University of Mining and Technology, Xuzhou, 221116 Jiangsu China; 2 School of Safety Engineering, China University of Mining and Technology, Xuzhou, 221116 Jiangsu China

**Keywords:** Acoustic emission, Brittleness, Rock, Damage model, Natural hazards, Solid Earth sciences

## Abstract

Due to the complexity of the strata, it is difficult to monitor and identify the disasters induced by rock fractures in the process of mining deep coal resources. This will seriously affect the safety and sustainable mining of coal. Therefore, it is necessary to understand the failure mechanisms and acoustic emission (AE) characteristics of different rocks. In this paper, uniaxial compression tests as well as simultaneous AE monitoring were carried out on four different rocks. The four rocks include yellow sandstone, white sandstone, marble and limestone. The mechanical properties, energy evolution and AE characteristics of different rocks were analysed. It is found that the AE response of rocks is closely related to the damage and fracture process. The more brittle the rock is, the less energy is dissipated before failure, and the less obvious the AE precursor is, and the *RA*-*AF* values can effectively characterise the failure modes of different rocks. Finally, the damage models were developed from the perspectives of AE energy and dissipated energy, respectively. The damage model based on dissipated energy can better reflect the stress and damage state of the rock, and the theoretical curves of stress–strain are in good agreement with the measured curves.

## Introduction

As coal resources mining gradually enter the deep stratum, engineering practice faces the challenge of high geo-stress. Rock fracture under high geo-stress often triggers serious engineering disasters, such as rock burst and large deformation^1–5^. This will seriously affect the safety and sustainable mining of coal. It is very important to understand the rock damage and fracture mechanism and carry out effective monitoring and early warning, which is also a research hotspot in the field of rock engineering. Acoustic emission (AE) is an elastic waveform signal generated by rock fracture, which contains rich fracture information^6^. This technique is widely used in monitoring and early warning of rock stability, both in the laboratory and at the engineering site^7–10^. However, achieving accurate monitoring and early warning of disasters is difficult because of the complexity of the stratigraphy and the variability of different rocks. Rocks are often composed of multiple minerals. The differences in mechanical properties of different rocks are essentially caused by the differences in their mineral components and microstructures. The strength of mineral particles, the bonding mode between particles, and the bonding strength are all factors that determine the mechanical properties of rocks. In-depth study of the differences in the mechanical behaviour of different rocks and the characteristics of AE signals is essential.

A great deal of previous research has been carried out on the damage and fracture mechanisms as well as the AE characteristics of loaded rocks. Kong et al.^11^ investigated the AE time series parameters as well as the frequency response characteristics of sandstone under uniaxial compression after different temperature treatments, and established the relevant damage constitutive model to explain the thermally induced rock damage mechanism. Wang et al.^12^ carried out uniaxial compression tests on fracture-bearing sandstones at different loading rates to study the AE response characteristics during crack propagation. Li et al.^13–15^ conducted uniaxial compression tests on sandstones with different water content to analyse the laws and mechanisms of water influence on the strength, energy evolution and AE response of rocks, and investigated the AE precursor characteristics of the failure of sandstones with different water contents. AE response and damage evolution characteristics of frozen sandstone under lateral unloading were investigated by Liu et al.^16^. Experiments to investigate the AE spectral characteristics of coal rock under hydraulic fracturing and uniaxial compression were carried out by Qian et al.^17^. To summarise, the current studies cover the AE properties under the influence of loading conditions, water and temperature, among other factors. These studies provide reference and inspiration for experimental methods, AE signal analysis methods, rock fracture and AE mechanisms. However, there are fewer studies comparing the brittleness, AE properties and damage evolution of different rocks, which is needed.

In this paper, in order to further understand the mechanism of rock damage and improve the accuracy of underground engineering disaster monitoring, considering the differences in rock properties, uniaxial compression testing and AE monitoring were conducted on four different types of rocks. The four types of rocks are yellow sandstone, white sandstone, marble, and limestone. The mechanical properties, energy evolution laws and AE signal characteristics of different rocks were analyzed. Finally, damage models were established from the perspective of AE and energy evolution to describe the stress–strain relationship of different rocks under load. The research results are conducive to ensuring the safety and sustainable mining of coal.

## Experimental materials and procedures

**Figure 1:**
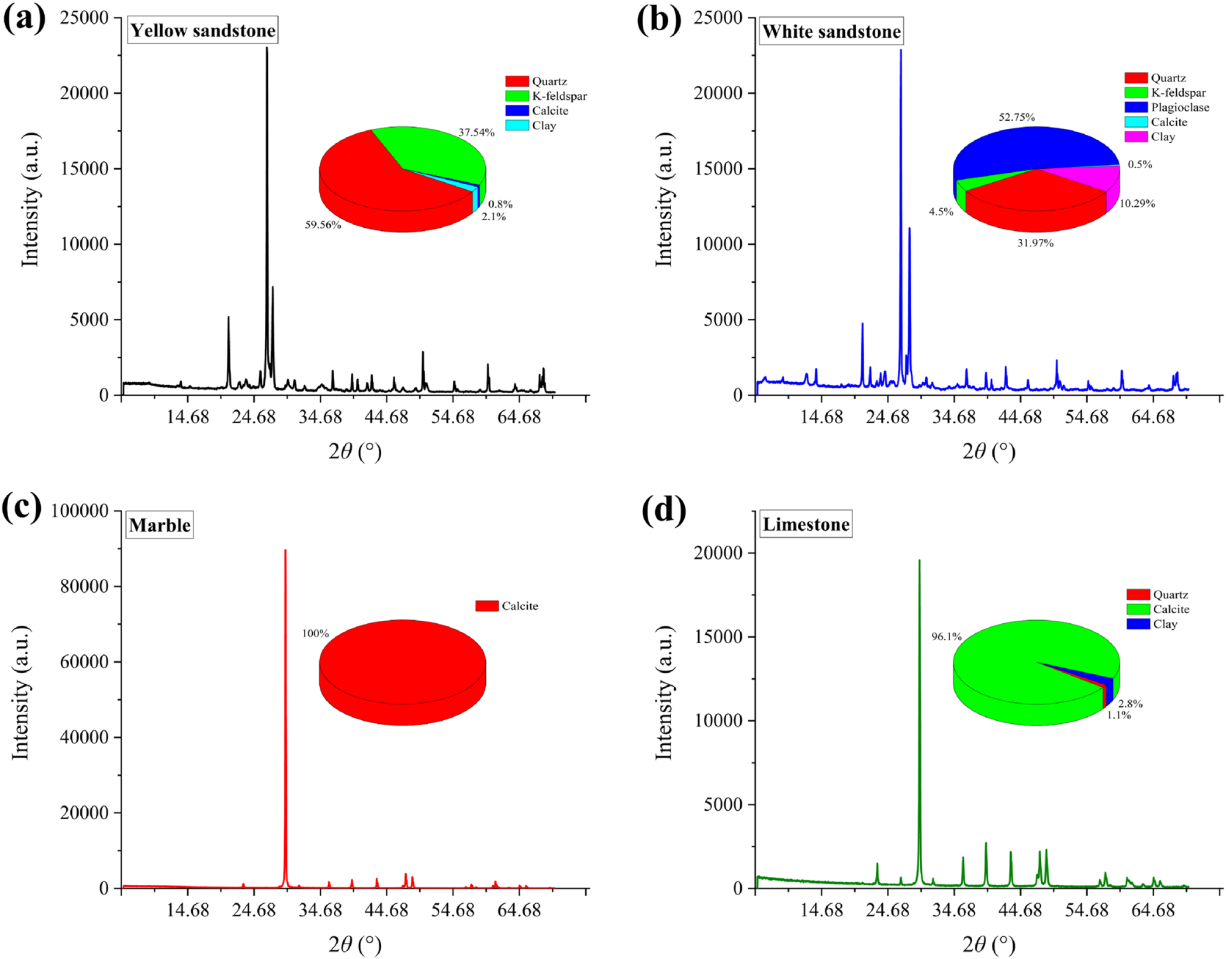
XRD spectrum: (**a**) Yellow sandstone; (**b**) White sandstone; (**c**) Marble; (**d**) Limestone.

Four different rocks were used to carry out the experimental study, they are white sandstone, yellow sandstone, marble and limestone. They were analysed for their mineralogical composition and percentage by X-ray diffraction (XRD) tests, and the XRD spectra obtained from the tests are shown in Fig. 1. The percentage of specific compositions as well as the density properties of the four rocks are listed in Table 1. All rocks were fabricated into cylinders with a height of 100 mm and a diameter of 50 mm for subsequent uniaxial compression mechanical tests according to the standards given by the International Society of Rock Mechanics (ISRM).

**Table 1:** Mineral components.

Rock types	Density (g/cm^3^)	Quartz (%)	K-feldspar (%)	Plagioclase (%)	Calcite (%)	Clay (%)
Yellow sandstone	1.8	59.5	37.5	0	0.8	2.1
White sandstone	2.7	32	4.5	52.8	0.5	10.3
Marble	3.1	0	0	0	100	0
Limestone	2.4	1.1	0	0	96.1	2.8

Uniaxial compression testing was carried out using the new SANS microcomputer-controlled electro-hydraulic servo pressure testing machine, which allows displacement and force to be controlled and collected with high precision. The loading rate is set to 500 N/s. AE signals were acquired simultaneously during the loading process. The AE signal acquisition system was the 24-channel Micro-II type AE monitoring host of American Physical Acoustics Corporation with a NANO-30 AE probe and a preamplifier. The AE probe was placed close to the surface of the specimen through the coupling agent, and the AE signals generated from the rock fracture captured by the probe were transmitted through the preamplifier to the host computer for further data analysis and storage. The threshold, preamplification and the acquisition frequency for AE acquisition were set to 40 dB, 40 dB and 2 × 10^6^/s, respectively. The press was switched on simultaneously with the AE acquisition until the specimens were completely destroyed. Three replicate experiments were conducted for each rock type, with a total of 12 samples tested. The sample number and mechanical test results are shown in Table 2.

**Table 2:** Specimen number and test results.

Rock types	Sample No	*USC* (MPa)		*E* (GPa)		*B*_*I*_	
		Value	Average	Value	Average	Value	Average
Yellow sandstone	YS-1	38.3	38.0	6.0	5.8	0.68	0.67
	YS-2	36.6		5.4		0.66	
	YS-3	39.1		6.2		0.68	
White sandstone	WS-1	69.8	70.1	11.3	11.3	0.87	0.88
	WS-2	68.3		10.3		0.89	
	WS-3	72.1		12.3		0.87	
Marble	M-1	52.3	51.6	13.3	13.3	0.62	0.60
	M-2	49.3		12.4		0.60	
	M-3	53.1		14.2		0.59	
Limestone	L-1	45.1	45.5	8.8	8.6	0.71	0.71
	L-2	48.2		8.1		0.70	
	L-3	43.2		9.0		0.71	

## Mechanical and energy characteristics

### Strength, deformation and brittleness

**Figure 2:**
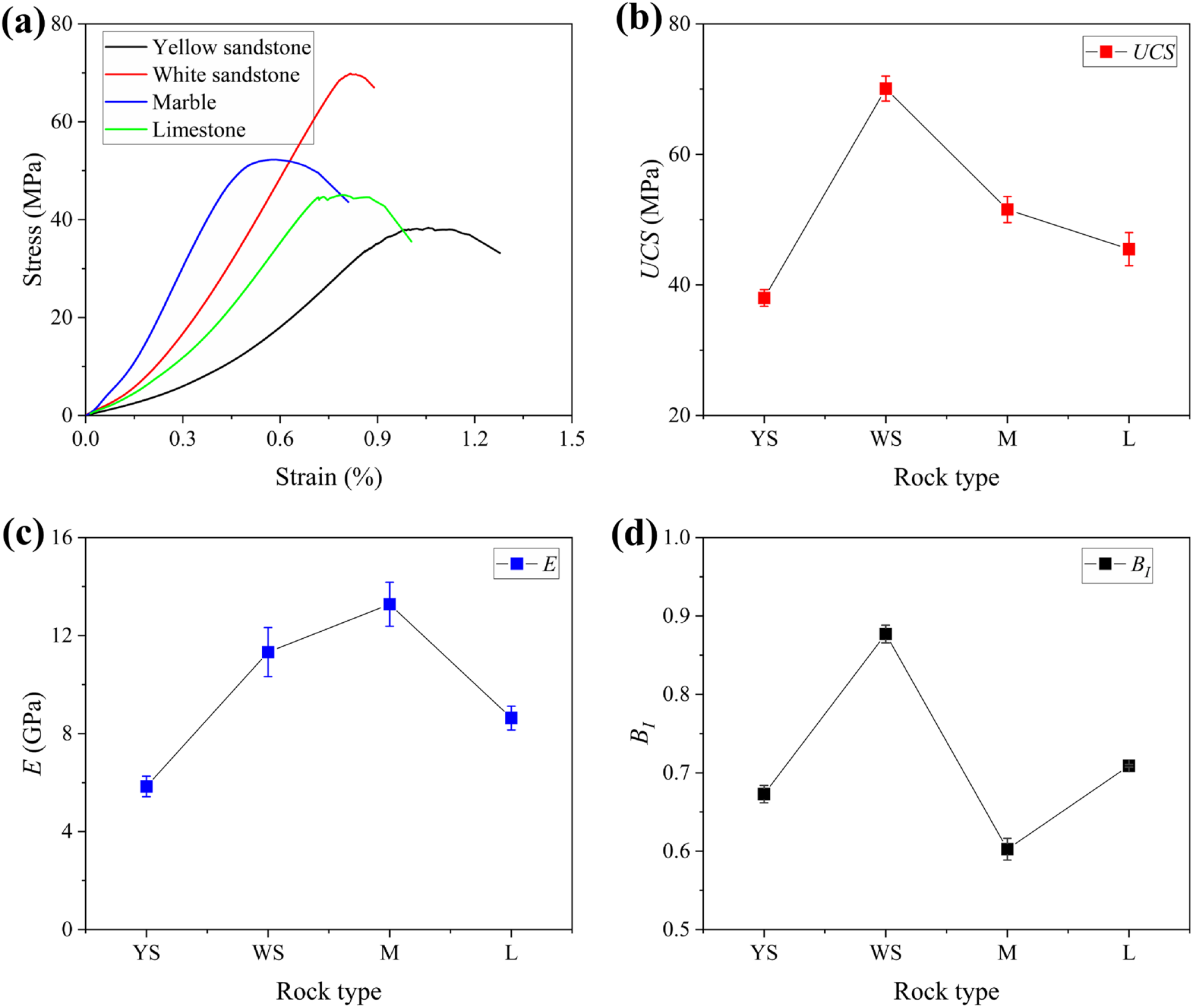
Mechanical properties: (**a**) stress–strain curves; (**b**) *USC*; (**c**) *E* and (**d**) *B*_*I*_. (YS, WS, M and L in the figure refer to yellow sandstone, white sandstone, marble and limestone respectively).

Figure 2 gives the test results of mechanical properties of different rocks under uniaxial compression conditions. Figure 2a shows the stress–strain curves of a set of typical specimens. Figure 2b–d show the uniaxial compressive strength *UCS*, elastic modulus *E* and brittleness index *B*_*I*_ of all the specimens, respectively. The brittleness index *B*_*I*_^18^ adopts a classical index based on the pre-peak energy storage property, which is calculated in the following equation:

1 $$B_{I} = U^{ep} /U^{p} = \frac{{UCS^{2} }}{2E}/\mathop \smallint \nolimits_{0}^{{\varepsilon^{p} }} \sigma d\varepsilon$$

where stress is σ and strain is ε, *U*^*ep*^ is the elastic energy stored at peak stress, *U*^*p*^ is the total amount of work done by the outside world at peak stress; *ε*^*p*^ is the strain at peak stress.

All the specimens went through the stages of compression, elastic deformation, crack propagation, and failure during the loading process. Specifically, the *UCS* of yellow sandstone, white sandstone, marble and limestone specimens averaged 38 MPa, 70.1 MPa, 51.6 MPa and 45.5 MPa, respectively, whereas the *E* were 5.8 GPa, 11.3 GPa, 13.3 GPa and 8.6 GPa, respectively. In terms of brittleness, white sandstone has the highest brittleness index; it is the most brittle. This means that white sandstone stored the vast majority of strain energy in the form of elastic energy in the specimen prior to peak stress, and then is destroyed suddenly upon reaching peak stress. Its failure was abrupt. In order of brittleness, the four rocks are: white sandstone, limestone, yellow sandstone, and marble.

### Laws of energy evolution

During the loading process, the press continuously works on the sample, and the total energy absorbed by the sample is *U*. A portion of the absorbed energy is stored in the sample in the form of elastic energy *U*^*e*^, while a portion *U*^*d*^ is dissipated due to rock fracture, irreversible deformation, and radiation signals (such as acoustic emissions). In order to further investigate the energy evolution law of different rocks during the loading process, the changes of their total strain energy *U*, elastic energy *U*^*e*^ and dissipation energy *U*^*d*^ during the loading process were calculated. The specific calculation equations are as follows^13,19^:

2 $$U={\int }_{0}^{\varepsilon }\sigma d\varepsilon$$

3 $${U}^{e}=\frac{{\sigma }^{2}}{2E}$$

4 $${U}^{d}=U-{U}^{e}$$

**Figure 3:**
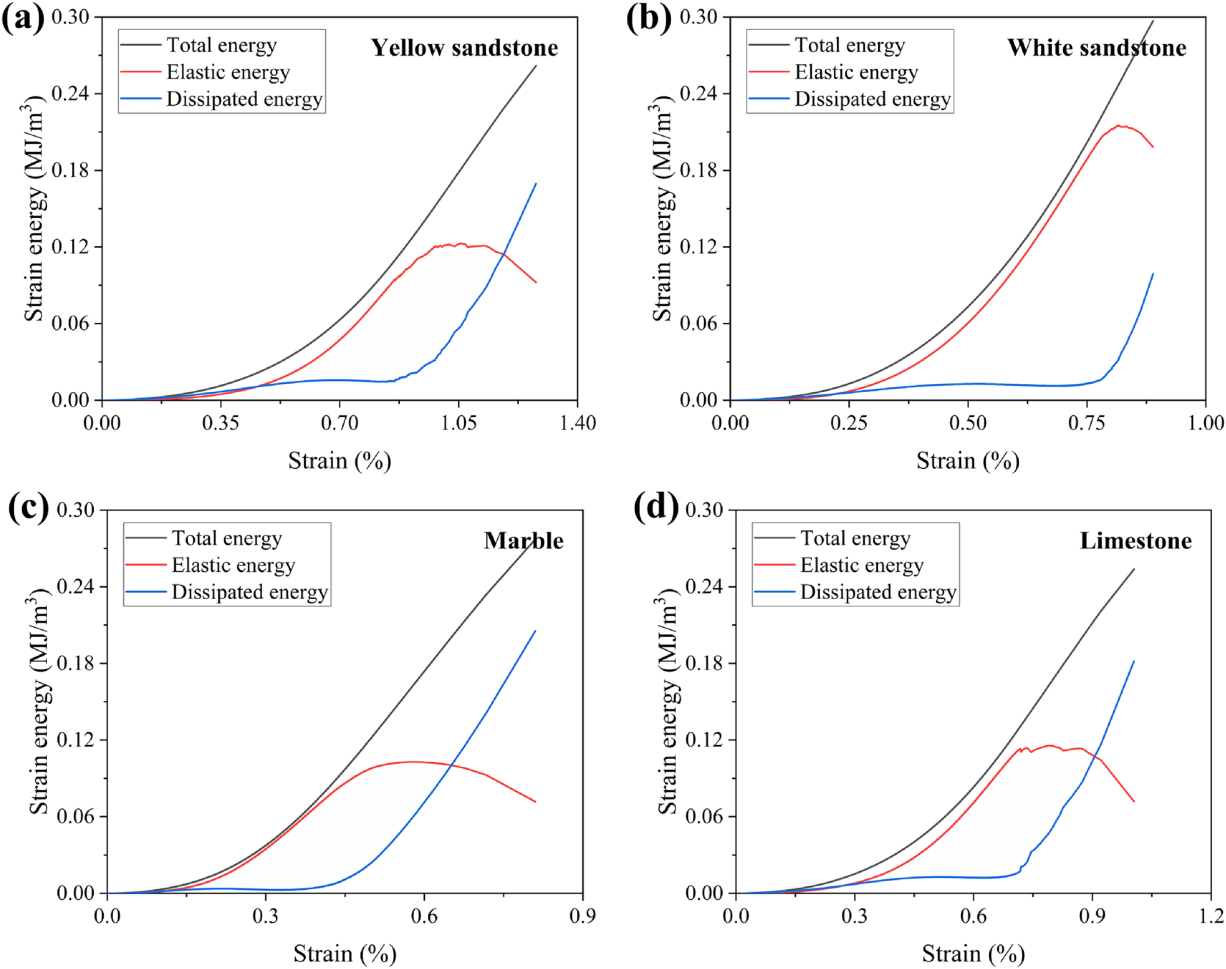
Laws of energy evolution: (**a**) Yellow sandstone; (**b**) White sandstone; (**c**) Marble; (**d**) Limestone.

Figure 3 shows the calculation results for one set of different rocks. The vast majority of the work done by the outside world during the pre-loading period is stored in the rock in the form of elastic energy, and only a small part of it is dissipated due to irreversible deformation and fracture. After the end of elastic deformation, cracks start to develop inside the specimen and the increase in dissipated energy is gradually significant. After the peak stress, the stored elastic energy in the sample reaches its limit and is released, causing the sample to be failed and the dissipated energy to sharply increase. As characterised by the brittleness indicator *B*_*I*_, the dissipated energy growth of the white sandstone before peak stress was not significant. In contrast, the weakest brittle marble showed a significant increase in dissipated energy during the crack propagation phase before the peak.

### Failure mode

**Figure 4:**
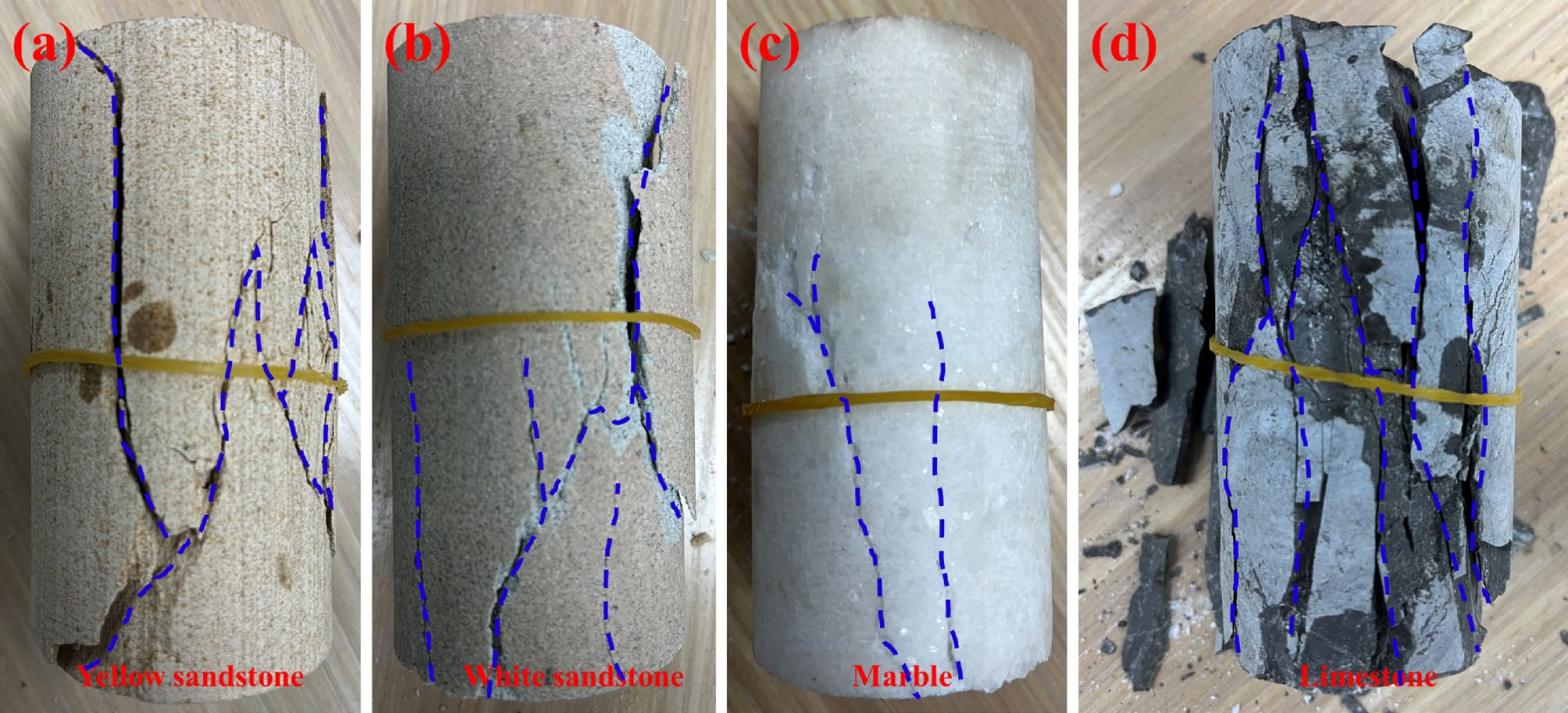
Failure mode: (**a**) Yellow sandstone; (**b**) White sandstone; (**c**) Marble; (**d**) Limestone.

Figure 4 illustrates the final failure mode of a group of different rocks. The blue lines in the figure mark the locations of some major cracks. As can be seen from the figure, the rupture of all samples under uniaxial compression conditions is dominated by tensile fracture (fracture parallel to the loading direction), accompanied by some shear fracture (fracture at an angle to the loading direction). There is a large tensile crack in the centre of the yellow sandstone, and a typical tensile crack is also present at the right edge, connected by some shear cracks. A significant tensile fracture occurred in the upper right part of the white sandstone, and a shear crack is present in the lower half as well as some small tensile cracks. Two mixed tensile-shear cracks are present in the lower half of the marble. The limestone is highly fragmented and is dominated by tensile fractures.

## AE response characteristics

### Time-series variation of AE energy

**Figure 5:**
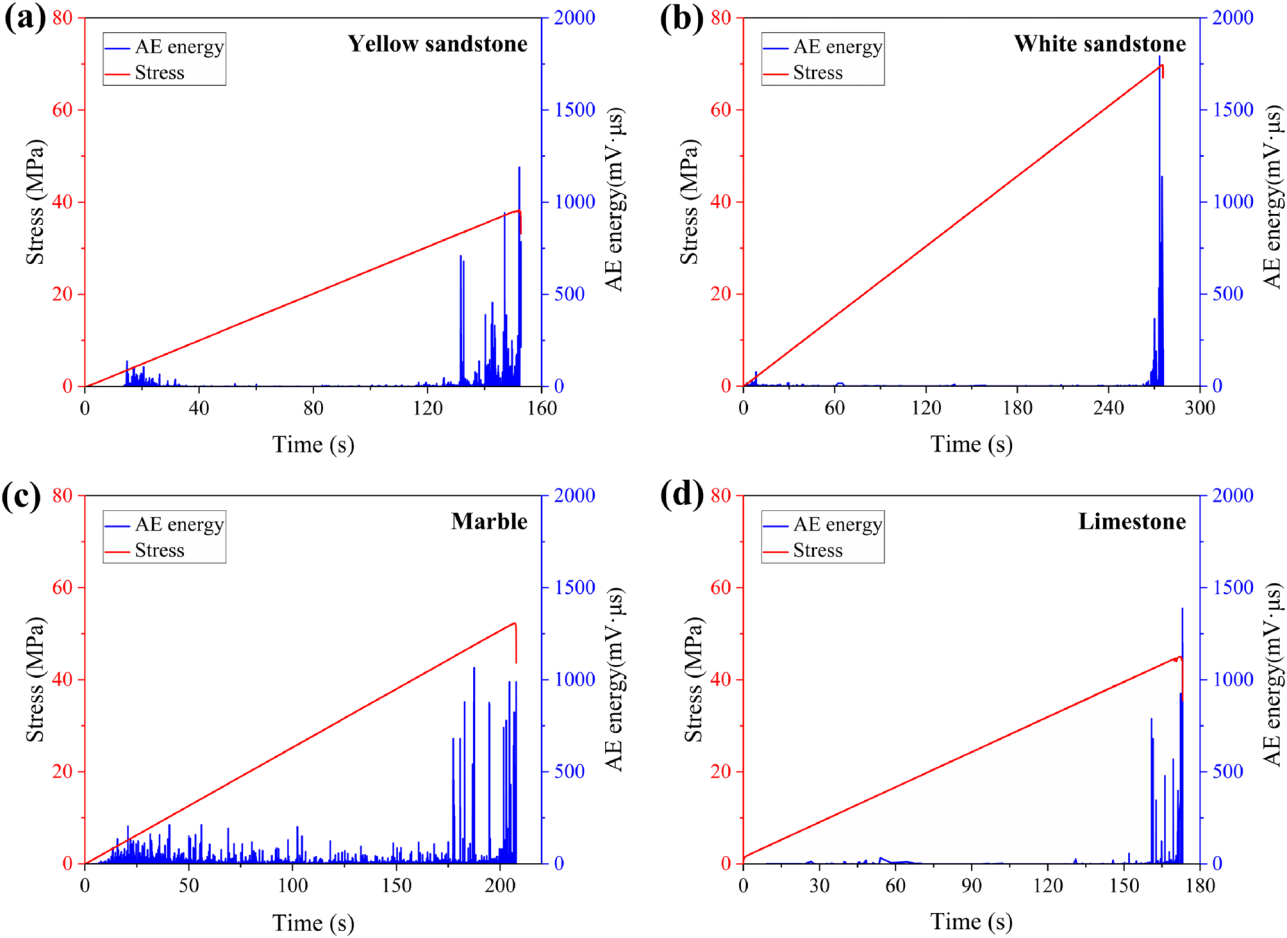
Temporal variation of AE energy: (**a**) Yellow sandstone; (**b**) White sandstone; (**c**) Marble; (**d**) Limestone.

**Figure 6:**
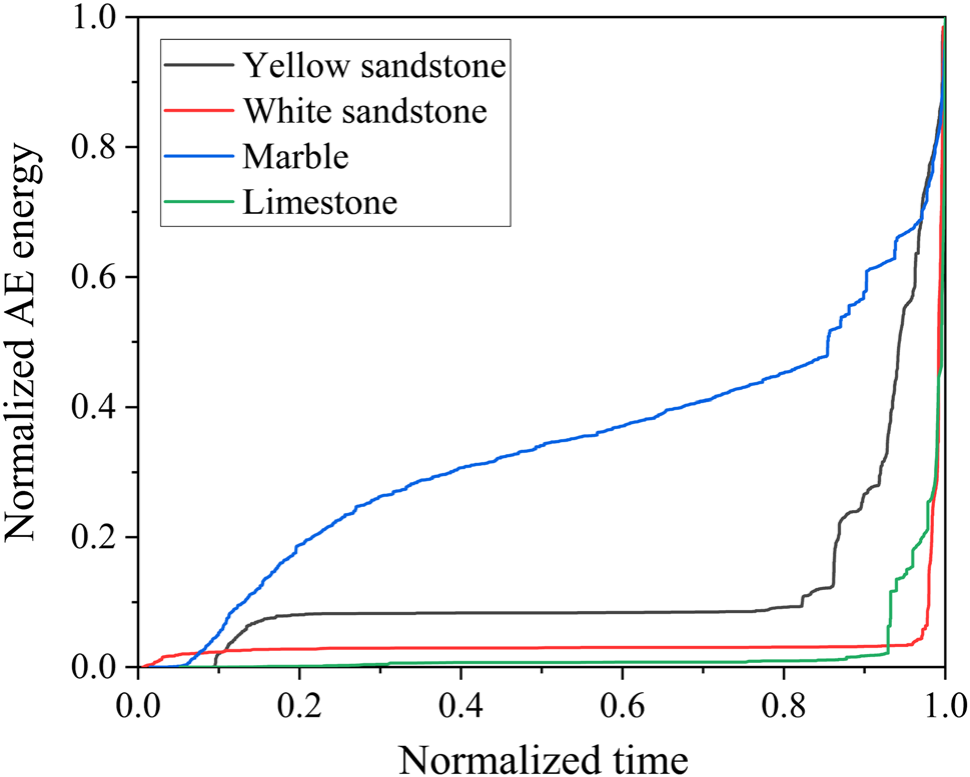
Normalised cumulative AE energy.

Figure 5 shows the temporal variation of the AE energy of a group of different rocks during the loading process. In the pre-loading stage, the specimen was basically not ruptured internally and the AE energy was maintained at a low level. Upon entering the crack propagation phase, the AE energy increased abruptly and tended to peak at the moment of failure. Changes in the AE energy are closely related to the processes of fracture, damage and energy dissipation within the specimen. Moreover, the more brittle the rock, such as white sandstone, the more sudden the increase in AE energy when failure and the less obvious the precursor. In the case of less brittle rocks, the large amount of energy dissipation during the pre-peak crack propagation phase leads to an increase in AE, which also leads to richer failure precursor information. Figure 6 normalises the evolution of the accumulated values of AE energy for this group of rocks. It can be clearly seen that the weaker the brittleness of the rocks, the earlier the growth of the AE energy in the later stages of loading.

### RA-AF value

**Figure 7:**
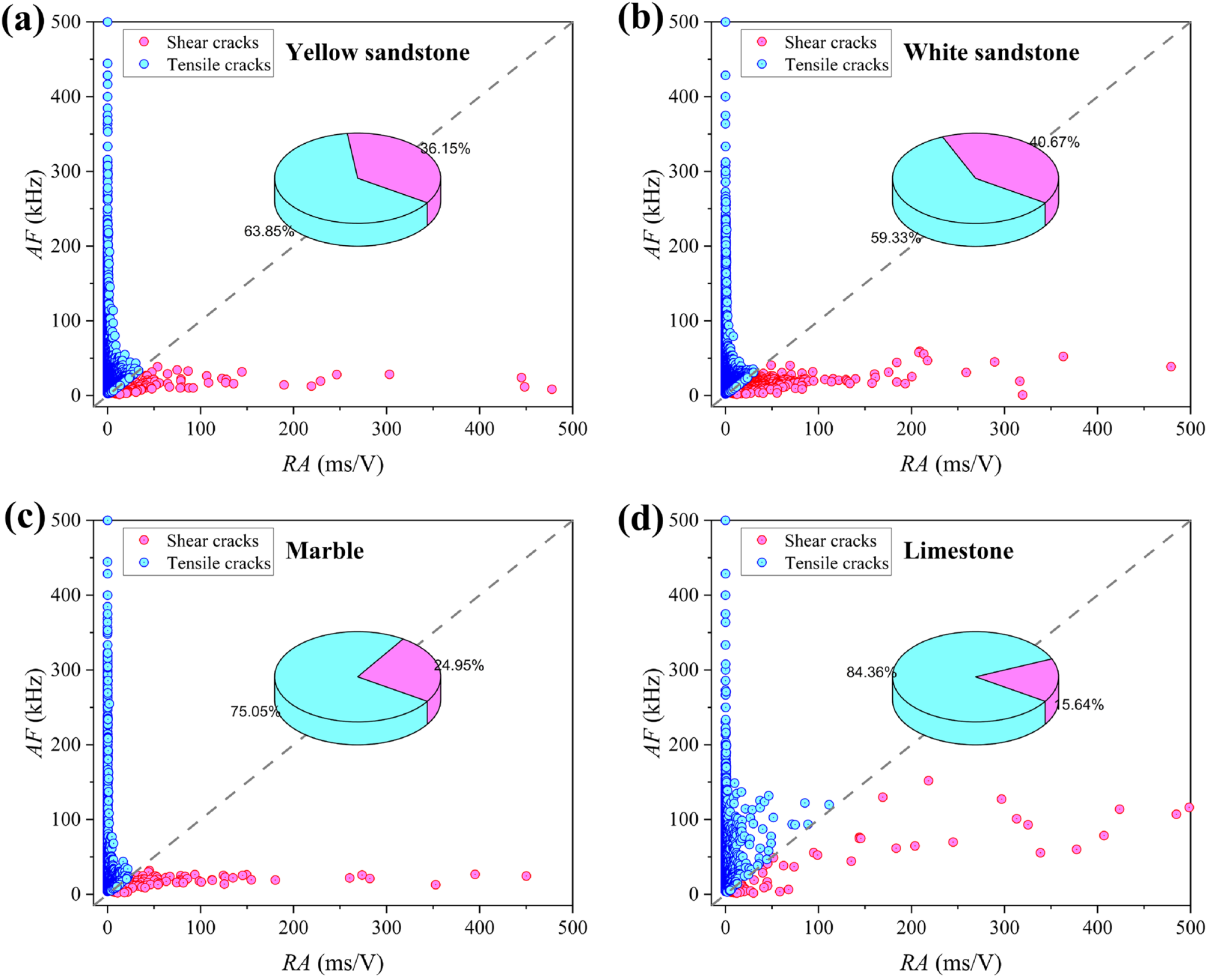
*RA*-*AF* value: (**a**) Yellow sandstone; (**b**) White sandstone; (**c**) Marble; (**d**) Limestone.

Tensile cracks produce AE signals with short waveforms, fast rise times and high frequencies, while the opposite is true for shear cracks. Therefore, cracks can be classified by analysing the AE parameters^20^. Average frequency (AF) and rise angle (RA) were defined and used as classification criteria. A ratio of AF to RA of 1 was used as the cut-off between tensile and shear fracture^21^. The *RA*-*AF* values for a set of different rocks are shown in Fig. 7. The percentage of shear cracks is 36.15%, 40.67%, 24.95% and 15.64% for yellow sandstone, white sandstone, marble and limestone respectively while the percentage of tensile cracks is 63.85%, 59.33%, 75.05% and 84.36% respectively. This is consistent with their failure patterns, with some shear fracture in the white and yellow sandstones, and minimal shear cracking in the limestone.

## Damage evolution model

According to the well-known principle of equivalent strain^22^, the damage constitutive model of rock can be written as the following equation:

5 $$\upsigma =E(1-D)\varepsilon$$

where *D* is the damage variable. At present, there are many ways to define the damage variable. For example, it is considered that the statistical distribution law of the damage produced by micro rupture and AE is the same, and the damage variable *D* is directly defined as^11^:

6 $${\text{D}}={\text{N}}/{N}_{m}$$

where *N* is the cumulative AE energy that increases with strain and *N*_*m*_ is the total AE energy.

Alternatively, take an energy perspective. Since rock damage is an energy-driven change of state, the process of asymptotic rock damage is a process of energy dissipation. Therefore the damage variable *D* can be defined as:

7 $${\text{D}}={U}^{d}/{{\text{U}}}^{dt}$$

where *U*^*d*^ is the dissipated energy that increases with strain and *U*^*dt*^ is the total dissipated energy required for complete failure.

Equations (6) and (7) were brought into Eq. (5) to obtain the damage constitutive models from the AE perspective and the energy perspective, respectively:

8 $$\upsigma =E(1-\frac{N}{{N}_{m}})\varepsilon$$

9 $$\upsigma =E(1-\frac{{U}^{d}}{{U}^{dt}})\varepsilon$$

**Figure 8:**
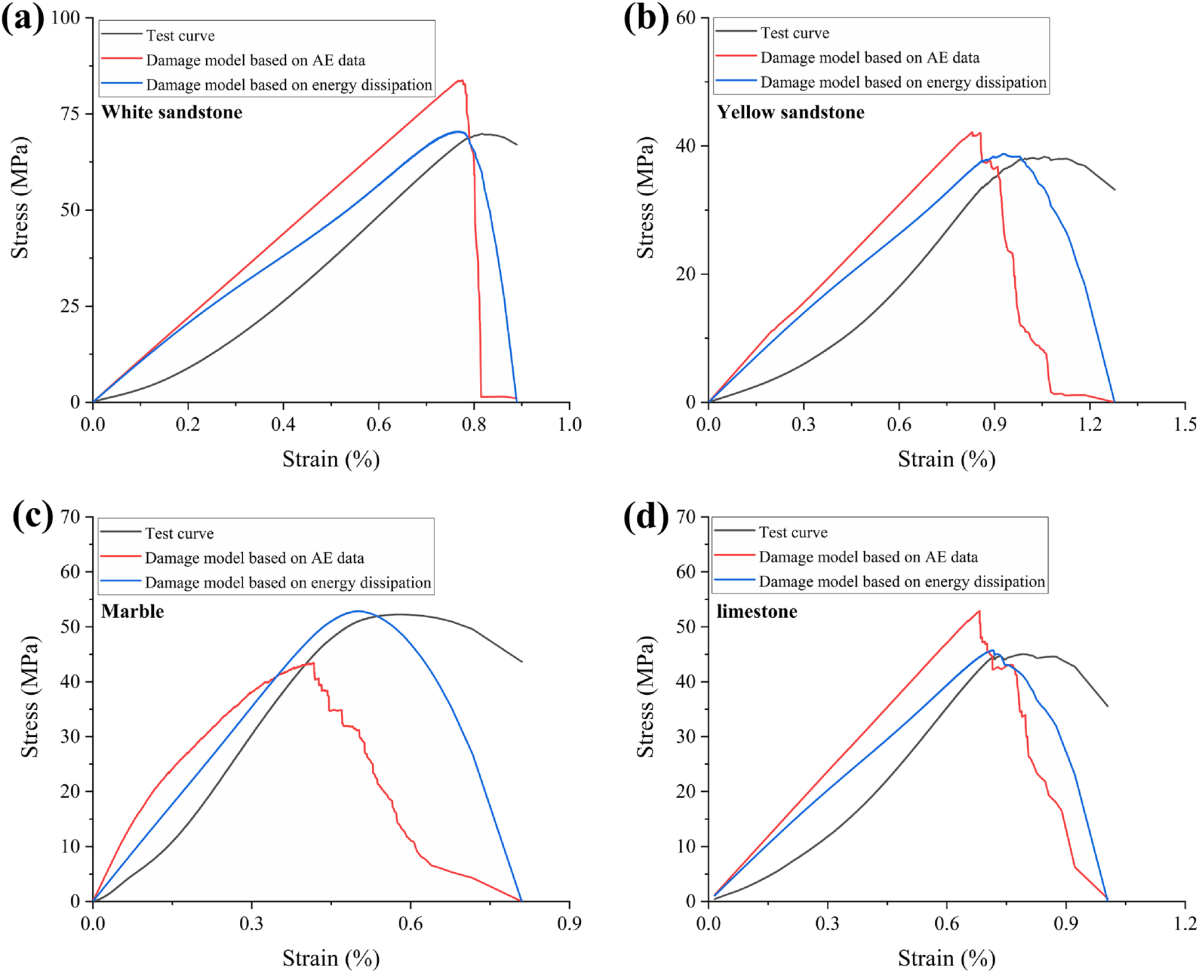
Comparison of damage model and experimental results: (**a**) Yellow sandstone; (**b**) White sandstone; (**c**) Marble; (**d**) Limestone.

Based on the above models, the stress–strain curves obtained from the calculations are shown in Fig. 8. In general, the overall trends of the theoretical and experimental curves are generally consistent. Both AE and dissipative energy based damage models can generally assess the stress state of the rock. However, it seems that the damage model based on dissipated energy performs better and is closer to the experimental curves. This may be due to the fact that AE only reflects brittle fracture within the rock, which is relatively one-sided. Dissipation energy reflects all irreversible state changes such as fracture as well as plastic deformation, and is a more comprehensive consideration.

## Conclusions and summary

In this paper, uniaxial compression tests were carried out on four different rocks. Moreover, simultaneous AE monitoring was carried out. The four rocks include yellow sandstone, white sandstone, marble and limestone. The strength, deformation, brittleness and failure modes of the different rocks were analysed. And, the temporal evolution of the AE energy with *RA*-*AF* values was explored. The uniaxial compression strength of yellow sandstone, white sandstone, marble and limestone specimens averaged 38 MPa, 70.1 MPa, 51.6 MPa and 45.5 MPa, respectively, whereas the elastic modulus were 5.8 GPa, 11.3 GPa, 13.3 GPa and 8.6 GPa, respectively. In order of brittleness, the four rocks are: white sandstone, limestone, yellow sandstone, and marble. The AE response of rocks is closely related to the damage and fracture processes. The stronger the brittleness of the rock, the less energy dissipation before failure, and thus the less obvious the precursor of AE. The *RA*-*AF* value can effectively characterize the fracture modes of different rocks. Finally, damage models are established from the perspectives of AE energy and dissipation energy. The damage model based on dissipative energy can better reflect the stress and damage state of rocks, and the theoretical stress–strain curve is in good agreement with the measured curve.

## Data Availability

The related data used to support the findings of this study are included within the article.
